# Symptoms of Lesion Involving the Excitomotor Region of the Cortex

**Published:** 1917-03

**Authors:** Jas. N. Brawner

**Affiliations:** Atlanta, Ga.


### Journal-Record of Medicine

Successor to Atlanta Medical and Surgical Journal, Established 1855
and Southern Medical Record, Established 1870

**OWNED BY THE ATLANTA MEDICAL JOURNAL COMPANY**

PUBLISHED MONTHLY

*Official Organ Fulton County Medical Society, State Examing Board, Atlanta, Birmingham and Atlantic Railroad Surgeon's Association, Chattahoochee Valley Medical and Surgical Association, Etc.*

A. W. STIRLING, M. D., C. M., D. P. H.; J. S. HURT, B. Ph., M.D.
GEO. M. NILES, M. D., W. J. LOVE, M. D., (Ala.); Associate Editors

E. W. ALLEN, Business Manager

**COLLABORATORS**

W. F. WESTMORELAND, M. D., General Surgery
F. W. McRAE, M. D., Abdominal Surgery
ALLEN H. BUNCE, Medical Societies
E. B. BLOCK, M. D., Diseases of the Nervous System
MICHAEL HOKE, M. D., Orthopedic Surgery
CYRUS W. STRICKLER, M. D., Legal Medicine and Medical Legislation
E. C. DAVIS, A. B., M. D., Obstetrics
E. G. JONES, A. B., M. D., Gynecology
ALLEN H. BUNCE, M. D., Pathology and Bacteriology
R. T. DORSEY, Jr., B. S., M. D., Medicine
L. M. GAINES, A. B., M. D., Internal Medicine
GEO. C. MIZELL, M. D., Diseases of the Stomach and Intestines
L. B. CLARKE, M. D., Pediatrics
EDGAR PAULLIN, M. D., Opsonic Medicine
THEODORE TOEPEL, M. D., Mechano Therapy
E. G. BALLENGER, M. D., Diseases of the Genito-Urinary Organs

VOL. LXIII. ATLANTA, GA., MARCH, 1917. NO. 12

### SYMPTOMS OF LESION INVOLVING THE EXCITOMOTOR REGION OF THE CORTEX.

Jas. N. Brawner, M. D., Atlanta, Ga.

In view of the fact that the majority of gross lesions in the brain, causing hemiplegia, are located in or near the internal capsule, and involving in addition to the pyramidal tracts, the corpus striatum, we are apt to lose sight of the fact that a total destruction of the motor region of the cerebral cortex will not cause a complete paralysis of the opposite side of the body. The reason for this is that a large proportion of our movements involve reaction circuits, located in the central ganglia, and do not involve the cerebral cortex at all, or to a very small degree. It is very probable that this fact is not sufficiently kept in mind by neurologists. It will be remembered that some twenty-five years ago Goltz succeeded in completely removing, during three successive operations, the whole cerebral cortex of a dog. This dog lived for a period of eighteen months, and was afterward killed, showing that the operation had been complete. Of course, in this operation the entire excito-motor regions of the cortex on both sides were removed. This dog was able to walk, growl, bark, and would avoid objects to a certain extent. When food was placed in its mouth it would eat ravenously. At times it would lie down, and apparently sleep· It could be aroused by loud noises in the adjoining room. This operation showed that many of the reaction circuits for ordinary movements in the dog exist below the cerebral cortex. During the past few months Franc and several others have succeeded in completely removing the cortex of monkeys, as well as in dogs. They not only removed the cortex on one side, but in some instances on both sides and after recovery from the shock of the operations, it was surprising to see the absence of paralysis of the muscles in the extremities. They were able to perform almost as well as before the operation those movements phylogenetically old, that is, those acquired at an early date in the history of the species. These monkeys could walk, stand alone on the hind extremities, swing by the arms, and react to a certain extent to noises, and would eat when food was placed in their mouths. All actions, however, requiring voluntary initiation were absent. In other words, the movements were confined to reflex actions, though many of them were of a highly complex nature.

Several months ago, in carrying out some experiments on the frontal lobes in dogs, I was surprised to find that after the complete extirpation of the entire frontal lobes on both sides, including all of the excito-motor area, the dogs were still able to walk, run and play nearly as well as they did before the operation. Immediately following the operation there was complete paralysis of all four extremities, due, no doubt, to shock, but this soon disappeared. This operation was performed on about four or five dogs, and the same result was obtained in all. A permanent defect, however, remained, the recognition of which, I think, is of vast importance. This defect showed itself by the dog's inability to properly perform conscious attentive movements. Those actions and movements requiring the dog's conscious attention could not be performed. As an illustration, one of the dogs which had been operated on for several months was able to walk and run perfectly, except that it would now and then stumble and fall. Taking the dog to a gully, over which was a small round log, I was surprised to see that it was absolutely unable to walk the log, due to the fact that the act required attentive movements to bring about the proper muscular adjustments. The minute the dog was on the log, it would immediately fall off, perfectly helpless. It would get up, go back and try again, and finally the dog recognized its inability to perform the feat, and gave up, standing at the end of the log, barking intensely, as much as to say, "it is impossible for me to do it." From this experiment it is plain that should a dog or a monkey have an embolism, tumor or cyst, involving and completely destroying the excito-motor region of the cerebral cortex of the brain, the animal would not have a complete paralysis, due to the fact that the majority of its movements are phylogenetically old, and involve only the reflex circuits of the central ganglia, medulla, and spinal cord.

The question arises, is this the case in man? It is very probable that a larger proportion of our activities and movements are represented in the cortex, or rather involve certain reaction circuits in the cortex, than in the lower animals, though at the same time, it is probable that a large percentage of our movements do not involve the excito-motor region. I think that this explains why areas of softening, cysts, and tumors, involving the excito-motor region, do not produce complete paralysis in the muscles on the opposite side of the body, and in those cases where tumors originate in the excito-motor region, and cause complete paralysis, it is very probable that the paralysis is largely due to pressure on the reaction circuits in the central ganglia. The same may be said in reference to cases of hemiplegia, due to hemorrhage in the internal capsule. If this is the case it will furnish us a valuable diagnostic point. Suppose, for instance, a patient should have an area of softening, involving the hand and arm region of the cortex on the left side of the brain, completely destroying same. We would expect to find not complete paralysis of the right arm, but a disturbance of the voluntary attentive movements, the movements of precision, as in playing the piano, or fingering a violin, or in adjusting a piece of machinery. Such a patient, however, would be able to make the grosser movements of flexion, extension, abduction and adduction. It is very probable that the movements most severely affected would be those of the fingers, as these are most frequently used in acts of precision.

701-2 Grant Bldg.

